# Effects of Prenatal Phthalate Exposure and Childhood Exercise on Maternal Behaviors in Female Rats at Postpartum: A Role of *Oxtr* Methylation in the Hypothalamus

**DOI:** 10.3390/ijms22189847

**Published:** 2021-09-12

**Authors:** Yi-Ju Lee, Hwai-Ting Lin, Muhammad Asad Chaudhary, Yi-Ching Lee, Dean-Chuan Wang

**Affiliations:** 1 Department of Sports Medicine, Kaohsiung Medical University, Kaohsiung 80708, Taiwan; lyr092124@gmail.com (Y.-J.L.); whiting@kmu.edu.tw (H.-T.L.); 2 Ph. D. Program in Biomedical Engineering, College of Medicine, Kaohsiung Medical University, Kaohsiung 80708, Taiwan; asadchaudhary97@gmail.com; 3 Department of Food and Beverage Services, Tainan University of Technology, Tainan 710302, Taiwan; t10045@mail.tut.edu.tw; 4 Department of Physiology, College of Medicine, Kaohsiung Medical University, Kaohsiung 80708, Taiwan; 5 Department of Medical Research, Kaohsiung Medical University Hospital, Kaohsiung 80708, Taiwan

**Keywords:** maternal behaviors, oxytocin, DNA methylation, di-(2-ethylhexyl)-phthalate, exercise

## Abstract

Both the detrimental effect of prenatal exposure to di-(2-ethylhexyl)-phthalate (DEHP) and the beneficial effects of physical exercise on brain functions have been reported. The oxytocin pathway has been implicated in the onset of maternal behaviors. Epigenetic modification of the oxytocin receptor gene (OXTR) through DNA methylation has been associated with the pathogenesis of neuropsychiatric disorders. The purpose of this study was to investigate the effects of prenatal DEHP exposure on oxytocin-regulated maternal behaviors and to examine the protective effect of exercise. Pregnant rats (F0) were fed with vehicle or DEHP during gestation and the offspring females (F1) were assessed for their maternal behaviors by pup retrieval test at postpartum. The results showed that reduced pup retrieval activities without significant alteration of stress responses were observed in the prenatally DEHP-exposed females. Prenatal DEHP exposure decreased the expressions of oxytocin, *Oxtr* mRNA, and oxytocin receptor, and increased *Oxtr* methylation in the hypothalamus of postpartum female rats. There were no significant effects of exercise on behavioral, biochemical, and epigenetic measurements. These results suggest that prenatal DEHP exposure has a long-term adverse effect on maternal behaviors; *Oxtr* hyper-methylation may be a potential epigenetic mechanism for this alteration, which cannot be prevented by physical exercise during childhood.

## 1. Introduction

The quality of maternal care has a significant influence on the offspring’s physiological and psychological development across many mammalian species [1]. The initiation of maternal behaviors relies on the functions of neurotransmitters and neuromodulators acting during postpartum. The neuropeptide oxytocin, produced in the hypothalamus, is implicated as a system supporting neurobehavioral adaptation during pregnancy, childbirth, and postpartum caregiving in several species [2]. In humans, oxytocin levels are related to a set of maternal bonding behaviors, including gaze, vocalization, positive affect, affectionate touch, and frequent checking of the infant [3,4]. In rodents, oxytocin administration induces maternal behaviors such as more efficient pup retrieval and pup grouping in the nest [5,6], whereas oxytocin antagonist treatment impairs maternal behaviors in postpartum rats [7]. There is a concurrent expression of the oxytocin receptor (OTR) in the hypothalamus [8]. Rat dams with higher levels of OTR expression display enhanced maternal behaviors [9,10], while blockade of OTR by antagonist and gene knockout reduces the frequency of pup retrieval and licking/grooming [11,12,13]. Collectively, these studies provide strong evidence that the central oxytocin pathway plays an important role in activating and coordinating maternal behaviors.

The expression of OTR is determined by not only heritable genetic variation but also by epigenetic modification of the oxytocin receptor gene (human: *OXTR*; rodent: *Oxtr*) [14,15]. One of the epigenetic biomarkers is DNA methylation, which refers to the covalent binding of a methyl group to a cytosine nucleotide in the DNA sequence. In mammals, DNA methylation mainly modifies the cytosine and guanine dinucleotides (CpG sites) and generally alters the transcriptional activity [16]. Hyper-methylation within the promoter of *OXTR* appears to be responsible for the majority of epigenetic silencing of *OXTR* transcription [17]. An animal study shows that *Oxtr* methylation level is negatively correlated with OTR expression in the hypothalamus [18]. In humans, hyper-methylation of *OXTR* is associated with decreased OTR expression in the temporal cortex of autism patients, implicating the epigenetic regulation of *OXTR* in the pathogenesis of neuropsychiatric disorders [19]. Similar correlations between *OXTR* methylation and behavior are also reported in a broad range of socioemotional dysfunctions in humans, such as schizoaffective disorders, attachment anxiety, and depression [20,21,22]. Therefore, the methylation level of *OXTR* has been suggested as a prediction of phenotypic variability of the oxytocin pathway as well as of general impairments of social behaviors [23].

In addition to oxytocin, brain-derived neurotrophic factor (BDNF) has been implicated in the regulation of maternal behaviors. BDNF is a member of the neurotrophin family and plays a significant role in neuronal survival, synaptic plasticity, learning, and memory [24]. Hypothalamic BDNF deficiency may decrease the expression of oxytocin mRNA and reduce the pup retrieval, suggesting that coordination of BDNF and oxytocin in the hypothalamic circuit is involved in the regulation of maternal behaviors [25]. Rat dams with a higher level of BDNF in the brain exhibit an increased frequency of maternal licking/grooming behavior, which is mediated by the mechanism of oxytocin-induced BDNF expression [26]. These studies imply the synergistic interaction of oxytocin and BDNF in the establishment and maintenance of maternal behaviors.

The attempt to modulate oxytocin and BDNF to improve social behavior has been achieved by physiological or pharmacological intervention. Physical exercise has been shown to significantly stimulate the release of oxytocin and BDNF in humans [27,28]. In rodents, physical exercise induces the secretions of oxytocin and BDNF in the brain, implying a mechanism that involves the modulation of behavioral outcomes induced by exercise [29,30]. Physical exercise has been identified as an effective method to improve the symptoms of anxiety, depression, and cognitive deficits in humans and animals [31,32]. Epigenetic studies suggest that physical exercise can reduce the methylation levels within the promotors of some exercise-induced genes in human skeletal muscle and rodent brain [33,34,35]. Overall, these results suggest that physical exercise can differentially modulate the expression of gene transcripts through epigenetic mechanisms.

Early-life exposure to endocrine-disrupting chemicals may exert latent and profound consequences for the organization and regulation of the hypothalamic neuroendocrine systems [36]. Plasticizer di-(2-ethylhexyl) phthalate (DEHP), used to soften polyvinyl chloride plastics in many commercial items, has been identified as an endocrine-disrupting chemical [37]. The routes of DEHP exposure are through inhalation, ingestion, and dermal contact in children and adults, as well as through placental and nursing transfers in developing fetuses and newborns [38,39]. The fetal developing hypothalamus is vulnerable to DEHP exposure. For example, perinatal DEHP exposure may alter hypothalamic–pituitary regulation, resulting in reproductive dysfunction and precocious puberty in young rats [40,41]. The relationship between early-life DEHP exposure and socioemotional disorders has been established in humans and rodents. Perinatal DEHP exposure may increase anxiety-like behaviors in adult rats by dysregulating the feedback mechanism in the hypothalamic–pituitary–adrenal axis, such as increased adrenocorticotropic hormone (ACTH) levels and decreased corticosterone levels under stressed conditions [42,43]. Perinatal exposure to phthalates exerts an impact on hypothalamic gene expressions and social interactions in rodents [44,45]. In humans, higher serum DEHP levels are found in the autism spectrum disorders, hyperactivity, and inattention groups compared to healthy children, suggesting that endocrine disruptors may have a role in the pathogenesis of autism and attention-deficit hyperactivity disorder (ADHD) [46,47]. Importantly, altered *OXTR* methylation levels have been found in autism and ADHD patients [19,48]. This suggests that *OXTR* methylation patterns are altered across neurodevelopmental disorders and may be correlated with common clinical outcomes.

In our previous study, both the detrimental effect of early-life DEHP exposure and the beneficial effect of physical exercise on anxiety-like behaviors were reported [43]. Because maternal exposure to early-life stress during development can impair maternal care later in life [49], it is very likely that environmental factors, such as DEHP and exercise, may exert opposing effects on maternal behaviors by their counteraction of anxiety-like behaviors. Therefore, prenatal DEHP exposure with subsequent childhood exercise presents health concerns and the effect of combining these factors on maternal behaviors should be determined. Considering the critical role of oxytocin in the regulation of maternal behaviors, the current study aimed to investigate the effects of prenatal DEHP exposure and/or childhood exercise on hypothalamic oxytocin functions. To examine this hypothesis, prenatally DEHP-exposed female rats were trained to exercise during childhood–adolescence followed by a pup retrieval test at postpartum. Finally, the functions of hypothalamic oxytocin were evaluated to reveal the possible mechanisms underlying the effects of DEHP exposure and/or exercise on maternal behaviors.

## 2. Results

### 2.1. Effects of Prenatal DEHP Exposure and Childhood Exercise on Anxiety-like Behaviors in Postpartum Dams

The elevated plus-maze was performed on postpartum day 8 to examine anxiety-like behaviors (Table 1). There were no effects of DEHP and exercise on the number of open arms entries, the number of close arms entries, and the proportion of open arms entries. Meanwhile, there were no effects of DEHP and exercise on the exploration time in the open arms and close arms, and the percent of the time in open arms exploration. These results suggest that stress responses were not affected by either prenatal DEHP exposure or childhood exercise in postpartum dams.

### 2.2. Effects of Prenatal DEHP Exposure and Childhood Exercise on Levels of BDNF, ACTH, and Corticosterone in Postpartum Dams

BDNF and stress-related hormones in the plasma and hypothalamus were analyzed by ELISA (Table 2). The results showed that there were no effects of DEHP and exercise on the levels of BDNF in plasma and the hypothalamus. Regarding the stress-related hormones, the results showed an effect of DEHP on the enhancement of plasma ACTH levels [F_(1, 44)_ = 5.011, *p* < 0.05, η^2^ = 0.102], while no effect of exercise was found. Additionally, there were no effects of DEHP and exercise on plasma corticosterone levels in postpartum dams. These results suggest that secretions of BDNF and corticosterone were not affected by prenatal DEHP exposure and childhood exercise in postpartum dams.

### 2.3. Effects of Prenatal DEHP Exposure and Childhood Exercise on Maternal Behaviors in Postpartum Dams

**Figure 1:**
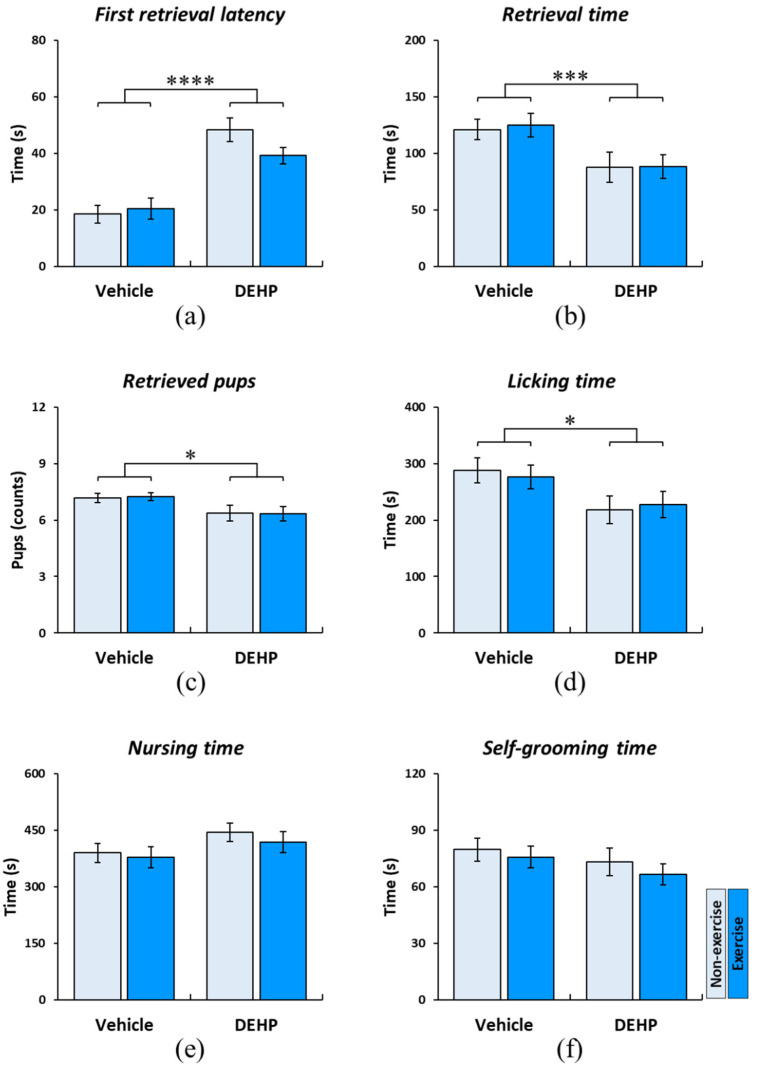
Effects of prenatal DEHP exposure and childhood exercise on maternal behaviors in postpartum dams. The pup retrieval test was performed postpartum, and the mean values of each responsive behavior were shown. There was a significant effect of DEHP on the first retrieval latency (**a**), the total retrieval time (**b**), the total number of retrieved pups (**c**), and the total licking time (**d**). No significant effects of DEHP and exercise on the total nursing time (**e**), and the total self-grooming time (**f**) were found in the present study. Data are presented in mean ± SEM (*n* = 12 in each group). *: *p* < 0.05; ***: *p* < 0.005; ****: *p* < 0.001.

There was a significant effect of DEHP on the first retrieval latency (F_(1, 44)_ = 48.607, *p* < 0.001, η^2^ = 0.525), while no effect of exercise was found (Figure 1a). This result revealed that DEHP-exposed dams began to retrieve their pups later than non-exposed females and exercise failed to ameliorate this impairment. A significant effect of DEHP was found on reducing the retrieval time in DEHP-exposed dams (F_(1, 44)_ = 10.255, *p* < 0.005, η^2^ = 0.189), suggesting that DEHP-exposed dams spent less time retrieving their pups. There was no effect of exercise on improving this impairment (Figure 1b). The number of retrieved pups was reduced by the effect of DEHP (F_(1, 44)_ = 7.111, *p* < 0.05, η^2^ = 0.139), while no effect of exercise was found (Figure 1c). There was a significant effect of DEHP on reducing the licking time (F_(1, 44)_ = 6.719, *p* < 0.05, η^2^ = 0.132), while exercise had no effect on ameliorating this reduction (Figure 1d). There were no effects of DEHP and exercise on nursing time and self-grooming time, suggesting that not only nursing behavior but also a few non-maternal behaviors were affected by DEHP and exercise (Figure 1e,f).

### 2.4. Effects of Prenatal DEHP Exposure and Childhood Exercise on the Oxytocin Pathway in Postpartum Dams

**Figure 2:**
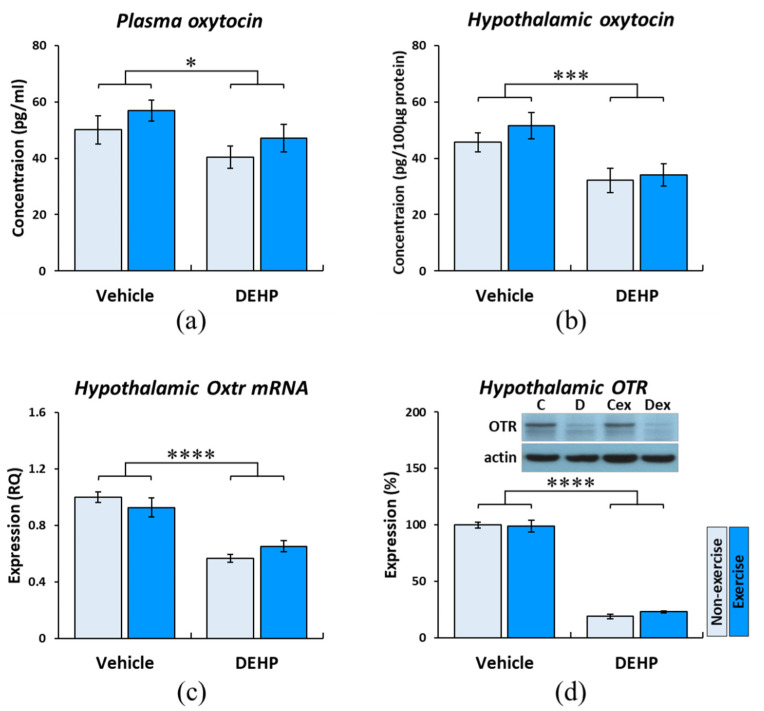
Effects of prenatal DEHP exposure and childhood exercise on the oxytocin pathway in postpartum dams. The results of ELISA showed that oxytocin levels in the plasma (**a**) and hypothalamus (**b**) were reduced by the effect of DEHP. (**c**) The result of qPCR showed a reduction of hypothalamic *Oxtr* mRNA influenced by the effect of DEHP exposure. (**d**) The result of the Western blot showed there was a significant effect of DEHP on reducing the hypothalamic OTR levels. Data are presented in mean ± SEM (*n* = 12 per group for plasma; *n* = 6 per group for hypothalamus). *: *p* < 0.05; ***: *p* < 0.005; ****: *p* < 0.001.

The results of ELISA showed that oxytocin levels in the plasma and hypothalamus were reduced by the effect of DEHP (plasma: F_(1, 44)_ = 4.759, *p* < 0.05, η^2^ = 0.098; hypothalamus: F_(1, 20)_ = 13.998, *p* < 0.005, η^2^ = 0.412), while no effect of exercise was found (Figure 2a,b). These results suggest that secretions of oxytocin were significantly reduced in the plasma and hypothalamus by prenatal DEHP exposure, and childhood exercise provided few effects on ameliorating this reduction in postpartum dams.

The expressions of hypothalamic *Oxtr* mRNA were quantified by qPCR. The result showed a significant reduction of *Oxtr* mRNA acting by the effect of DEHP exposure (F_(1, 20)_ = 59.145, *p* < 0.001, η^2^ = 0.747). However, no effect of exercise was obtained (Figure 2c). A significant effect of DEHP on reducing the expression of hypothalamic OTR was obtained by Western blot (F_(1, 20)_ = 643.977, *p* < 0.001, η^2^ = 0.970); there was also no effect of exercise on improving the expression of hypothalamic OTR (Figure 2d).

### 2.5. Effects of Prenatal DEHP Exposure and Childhood Exercise on Oxtr Methylation Levels in Postpartum Dams

**Figure 3:**
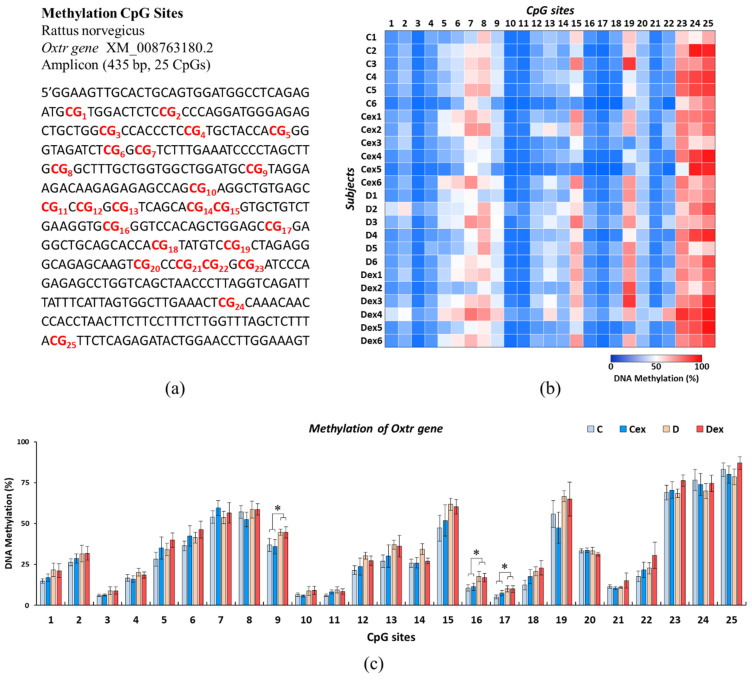
Effects of prenatal DEHP exposure and childhood exercise on *Oxtr* methylation levels in postpartum dams. (**a**) Epigram of 25 CpG sites (red-coded) within the *Oxtr* promoter region. (**b**) Heatmap of methylation levels of observed animals. (**c**) Mean methylation levels across 25 CpG sites in the hypothalamic *Oxtr* gene. The analyzed results revealed that 3 out of 25 CpG sites were significantly hyper-methylated by the effect of DEHP, including CpG9, CpG16, and CpG17. Data are presented in mean ± SEM (*n* = 6 in each group). *: *p* < 0.05.

DNA methylation was measured across a 435 base pair region of the *Oxtr* promoter spanning 25 CpG sites (Figure 3a). The methylation of each CpG site was analyzed by pyrosequencing. The overall methylation of each CpG site within the *Oxtr* gene was illustrated in Figure 3b.

Mean methylation levels across 25 CpG sites in the hypothalamic *Oxtr* gene were shown in Figure 3c. The analyzed results revealed that 3 out of 25 CpG sites were significantly hyper-methylated by the effect of DEHP, including CpG9 (F(_1, 20)_ = 5.559, *p* < 0.05, η^2^ = 0.217), CpG16 (F_(1, 20)_ = 6.118, *p* < 0.05, η^2^ = 0.234), and CpG17 (F(_1, 20)_ = 4.693, *p* < 0.05, η^2^ = 0.190), while there was no effect of exercise on *Oxtr* methylation across 25 CpG sites. This result suggested that methylation of the *Oxtr* gene was increased by prenatal DEHP exposure, and exercise during childhood had few effects on ameliorating this *Oxtr* hyper-methylation in postpartum dams.

### 2.6. Correlations between Oxtr Methylation, Oxtr Expression, and Maternal Behaviors

**Figure 4:**
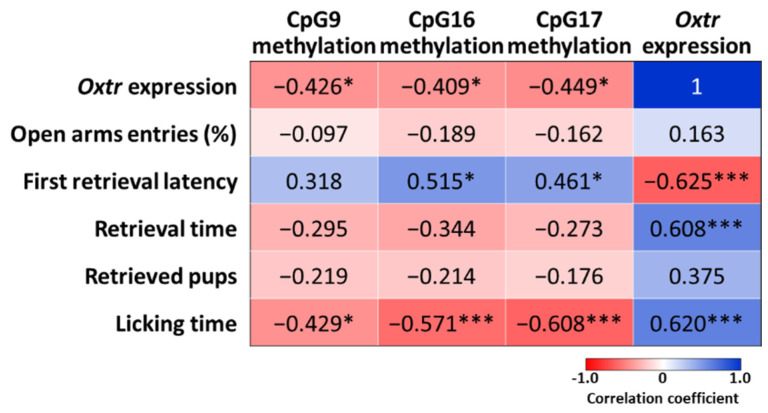
Correlations between *Oxtr* methylation, *Oxtr* expression, and maternal behaviors. Pearson’s correlation was used to assess the relationships between *Oxtr* methylation, *Oxtr* mRNA, and maternal behaviors. Data are presented in the correlation coefficient. *: *p* < 0.05; ***: *p* < 0.005.

Pearson’s correlation was used to assess the relationships between *Oxtr* methylation, *Oxtr* mRNA, and maternal behaviors. The correlation coefficients across 25 CpG sites are shown in Supplementary Materials Table S1, and data from CpG9, CpG16, and CpG17 are shown in Figure 4 for their significant vulnerability to prenatal DEHP exposure. The analysis showed that the *Oxtr* expressions were negatively correlated with *Oxtr* methylation levels in CpG9 (r = −0.426, *p* < 0.05), CpG16 (r = −0.409, *p* < 0.05), and CpG17 (r = −0.449, *p* < 0.05). Regarding maternal behaviors, the expressions of *Oxtr* mRNA were negatively correlated with the first retrieval latency (r = −0.625, *p* < 0.005), and positively correlated with retrieval time (r = 0.608, *p* < 0.005) and licking time (r = 0.620, *p* < 0.005). Furthermore, these three CpG sites were also significantly correlated with maternal behaviors, such as licking time, were negatively correlated with methylation levels in CpG9 (r = −0.429, *p* < 0.05), CpG16 (r = −0.571, *p* < 0.005), and CpG17 (r = −0.608, *p* < 0.005).

## 3. Discussion

The major finding of the present study is that prenatally DEHP-exposed female rats exhibited impaired maternal behaviors in some categories, such as longer latency to retrieve the first pup, lower number of retrieved pups, and less time spent in pup retrieval and licking. These results suggest that prenatal DEHP exposure had long-term effects on maternal behaviors in adulthood. However, a previous study reports that there is little influence of prenatal DEHP exposure on maternal behaviors in mice [45]. This disagreement may be caused by methodological differences, such as the administered dose and timing, as well as the duration of exposure. In Quinnies’ article, DEHP at doses of 5, 40, and 400 μg/kg/day were treated throughout pregnancy and during the first ten days of lactation, while we fed pregnant rats with DEHP (10 mg/kg/day) from gestational days 14 to 21. The effect of prenatal DEHP exposure on hypothalamic neuroendocrine function has been suggested as a non-monotonic dose-response profile with a J-shaped curve [50]. Prenatal DEHP exposure at higher doses (10–50 mg/kg/day) significantly interferes with the secretion of hypothalamic neurotransmitters and the expression of genes related to social behaviors [51,52]. This evidence suggested that the effect of prenatal DEHP exposure was dependent on the administered dose and the developmental stage of the hypothalamus. 

The alteration in the caregiving environment produces long-term changes in anxiety-related and social behaviors. Female rats who experienced low maternal care in their early life may demonstrate less maternal care for their offspring [53]. It is possible that gestational DEHP treatment might affect the quality of maternal caregiving in the F0 dams and transmit this dysregulation to the F1 offspring. Although the performance of maternal behaviors in the F0 dams is absent in the present study, previous evidence shows that gestational DEHP exposure has few effects on maternal behaviors in the DEHP-exposed F0 dams [44,45]. The poor maternal care experience may not or partially contribute to the disrupted maternal behaviors obtained in the prenatally DEHP-exposed female rats.

The hypothalamus begins to release oxytocin prohormone at embryonic day 14 in rodents. It is very likely to be the critical stage for the vulnerability of endocrine-disrupting effects [54]. This notion was supported by a reduction in hypothalamic gene expression in adult rats after DEHP exposure from gestational day 14 to 19 [52]. In the present study, the reductions in plasma and hypothalamic oxytocin, as well as hypothalamic *Oxtr* mRNA, and OTR, were found in the DEHP-exposed rats at postpartum, suggesting a long-term interference in oxytocin pathways caused by prenatal DEHP exposure. The oxytocin pathways play important roles in maternal behaviors; for example, oxytocin or OTR knockout animals exhibit a lower frequency of pup retrieval and pup licking [11,12]. Therefore, we showed that the reduction of the oxytocin pathway in the hypothalamus might underlie the impairment of maternal behaviors after prenatal DEHP exposure. To our knowledge, there is no information available on the disrupting activity of DEHP affecting oxytocin pathways linked to maternal behaviors. 

Several studies suggest that prenatal exposure to DEHP might induce developmental toxicity and endocrine disruption by altering DNA methylation in several tissues, such as the liver, blood cells, heart, and brain [55,56,57]. However, few studies have reported the epigenetic modification of prenatal DEHP exposure on the hypothalamic *Oxtr* gene. In the present study, increased hypothalamic *Oxtr* methylation in CpG9, CpG16, and CpG17 sites was found in prenatally DEHP-exposed female rats. There was a negative correlation between *Oxtr* methylation and *Oxtr* mRNA expression, suggesting hyper-methylation of the *Oxtr* gene might underlie the down-regulation of *Oxtr* mRNA in DEHP-exposed female rats. Evidence shows that *Oxtr* hyper-methylation in CpG5, CpG14, CpG15, and CpG25 sites are found in the peripheral blood mononuclear cells of female rats performing high-licking behaviors. However, *Oxtr* hyper-methylation in CpG6 and CpG7 sites is obtained in the hippocampus of low-licking dams [18]. In prairie voles, early life experience such as less parental care can increase *Oxtr* methylation in CpG18, CpG19, and CpG20 sites in the nucleus accumbens [58]. Interestingly, specific CpG sites are differentially methylated between distinct brain regions expressing different levels of *Oxtr* mRNA in mice brains [59]. This evidence suggests that brain region-specific methylation of the *Oxtr* gene may represent the effects of different environmental factors on epigenetic modification. In rodents, hyper-methylation of the *Oxtr* gene in the promoter region is associated with reduced *Oxtr* gene expression [18,58]. Some CpG sites within transcription factor estrogen receptor (ER) binding sites are sufficient to predict *Oxtr* mRNA expression [59]. Interestingly, perinatal DEHP exposure has been shown to reduce ER expression in adult rats’ hypothalamus and pituitary glands [52,60]. These studies suggest that prenatal DEHP exposure may impair the expression of *Oxtr* mRNA by concurrent alterations of *Oxtr* methylation and ERα functions.

The relationship between the environment, epigenetic modification, and behavior contributes novel findings of physical interaction of the environment with genes, leading to changes in behavior and health. Early-life adverse experiences, such as maltreatment, stress, and environmental toxicants, have been identified to affect gene expression by epigenetic modification [61]. The methylation of the *OXTR* gene has attracted considerable attention in the research of inter-individual differences in maternal behavior and social cognition [17,20]. In the present study, the *Oxtr* methylation levels were positively correlated with first retrieval latency but negatively correlated with licking time in prenatally DEHP-exposed female rats. This new evidence provides the causal link between prenatal DEHP exposure, *Oxtr* methylation, and maternal behaviors. Environmental factors, such as early-life stress and poor maternal care, have been identified to decrease *Oxtr* mRNA expression in the amygdala and hypothalamus of female rats [62,63]. In humans and rodents, higher levels of maternal care during childhood are associated with increased *OXTR* methylation within the promoter region in females but not in males [58,64,65]. Regarding *OXTR* methylation and behavior, lower levels of *OXTR* methylation and higher plasma oxytocin levels are associated with less socioemotional anxiety in young adults [21]. Interestingly, early-life stressed female rats give less care to their offspring. However, the administration of an epigenetic inhibitor can reduce the levels of adverse care toward their offspring by normalizing gene expressions [66]. Taken together, we investigated the relationship between *Oxtr* methylation, *Oxtr* mRNA expression, and maternal behaviors. The findings suggest that the alteration of *Oxtr* methylation may be an important mediator for down-regulation of the oxytocin pathway and subsequent maternal behavior deficiencies in prenatally DEHP-exposed female rats.

In the present study, prenatal DEHP exposure had few effects on anxiety-like behaviors in postpartum dams. We also reported that plasma ACTH levels were increased in the DEHP-exposed rats, while corticosterone levels showed no significant difference. Previous studies have shown that prenatal DEHP exposure may increase anxiety-like behaviors in adolescent animals [42,43]. Our current findings provide new evidence suggesting that emotional disturbance during adolescence might recover postpartum. The postpartum anxiolytic effect of oxytocin may have mediated the recovery of corticosterone levels and stress response in prenatally DEHP-exposed rats. The oxytocin system is functionally connected to the hypothalamic–pituitary–adrenal (HPA) axis interactively, such that the release of hypothalamic oxytocin during lactation provides an anxiolytic effect on stress-induced responses [67,68]. This buffering effect of oxytocin on stress response is important for the suppression of HPA reactivity to protect the fetus from adverse programming by maternal stress [69,70]. Importantly, evidence demonstrates that female rats with variations in maternal behavior, such as high-licking and low-licking rates, do not show behavioral differences in the elevated plus maze, the forced swimming test, or the open field test [71]. Therefore, it is very possible that enhanced oxytocin release at postpartum might provide an anxiolytic effect to buffer the stress response in the DEHP-exposed female rats.

The present study showed that there was no effect of prenatal DEHP exposure on plasma and hypothalamic BDNF levels in adult female rats at postpartum. Our result was in agreement with the effects of DEHP exposure on BDNF levels in a sex-specific manner, namely a reduction in males and preservation of BDNF in females [72,73]. The relationship between BDNF and oxytocin has been noticed; lower BDNF and oxytocin levels are correlated to the impairment of maternal behaviors and the symptoms of postpartum depression, suggesting coordination of BDNF and oxytocin in the regulation of behavioral outcomes at postpartum [25,26,74,75]. Early life stress strongly modulates BDNF and OTR expressions by the hyper-methylation of *BDNF* and *OXTR* genes in adult animals and humans, suggesting a synergism of BDNF and oxytocin in response to environmental insults [65,76]. The synergistic effect of BDNF and oxytocin was not observed in the present study, which was in agreement with the finding showing that both oxytocin and BDNF levels were higher in pregnant women. However, not oxytocin but BDNF levels were markedly decreased before and after childbirth [77,78].

In the present study, childhood exercise failed to recover the impaired oxytocin pathway and maternal behaviors in prenatally DEHP-exposed female rats at postpartum. We are concerned about the efficacy of the exercise regimen used in the present study. In male rats, prolonged voluntary wheel running results in a decrease in pituitary oxytocin content without evident changes in hormone concentrations in peripheral blood [79]. Forced swimming-induced oxytocin release is found in the hypothalamus without significant changes in plasma [30]. Although there were no significant changes in plasma and hypothalamic oxytocin in the exercised female rats of the present study, high levels of oxytocin release in the lactating dams may mask the effect of exercise-induced responses. The same exercise protocol has been identified to effectively ameliorate the cognitive and emotional deficits in DEHP-exposed adolescent rats [43,80,81]. Therefore, the efficacy of the exercise regimen may not be the factor interfering with the outcomes. Another noticed effect is the sex-specific manner of the exercise-induced BDNF response. The enhancement of BDNF is the most important mechanism underlying the improvement of cognitive function after exercise. However, evidence also shows that exercise-induced BDNF release is lower in females relative to males [82,83]. It is unlikely that sex difference is a key modulator of exercise effect, as our previous report indicates that exercise provides an anxiolytic effect on DEHP-exposed adolescent female rats [43]. It is possible that tissue or neuronal specific responses to exercise might affect the outcomes. Treatment of androgenic steroids may increase anxiety-like behaviors in female mice, while exercise does not ameliorate steroid-induced anxiety or alter amygdalar stress reactivity in females [84]. Evidence also shows that androgenic steroids impair hippocampal spatial learning and memory, and this effect is not rescued by exercise in male rats [85]. These results suggest that exercise is unable to improve the disruption of cognitive and emotional functions by androgenic steroid treatment depending on the investigated tissue-specific behaviors.

In conclusion, the present study provides evidence showing that prenatal DEHP exposure has a long-term adverse effect on maternal behaviors in adulthood. Some of these changes in behaviors may be associated with epigenetic modification of the *Oxtr* gene. Physical exercise during childhood provides a few effects on ameliorating epigenetic modification. Future work should combine detailed behavioral measures with analyses of epigenetic markers to establish a direct link between maternal emotions and caregiving following prenatal DEHP exposure, as well as to reveal the effects of exercise on ameliorating the socioemotional problem. As more research aims to understand the role of epigenetic markers on a range of human outcomes, it will be vital to have a comparable animal model. Our findings suggest that early-life exposure to endocrine disruptors will be useful in this regard, allowing for an expanded understanding of the role of epigenetic markers in controlling oxytocin pathways and impacting complex socioemotional behavior that can then inform and guide work on human conditions.

## 4. Materials and Methods

### 4.1. Animals

Sprague Dawley rats (BioLasco, Taipei, Taiwan) were used in the experiment. This study was carried out in strict accordance with the recommendations of the Guide for the Care and Use of Laboratory Animals of the National Institutes of Health. All the experimental procedures were approved by the Animal Care and Use Committee of Kaohsiung Medical University (IACUC Approval Number: 104160 approved on 4 May 2016), and all efforts were made to minimize the suffering and number of animals used.

### 4.2. Experimental Design

**Figure 5:**
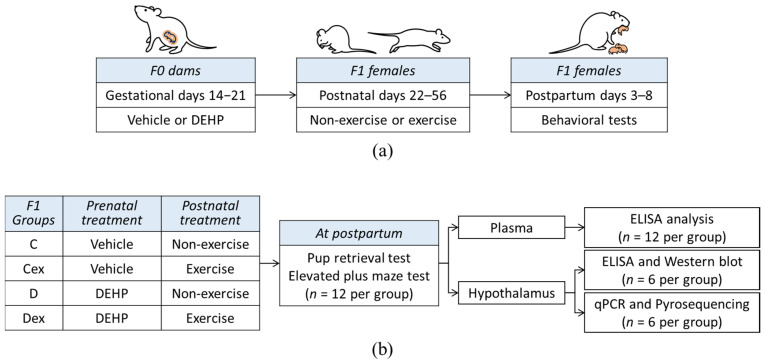
Schematic representation of the experimental design. (**a**) The experimental processes in F0 and F1 generations of dams. (**b**) Second-generation (F1) female rats were divided into four groups: vehicle control (c), exercised vehicle (Cex), DEHP exposure (d), and exercised DEHP exposure (Dex). The performances of maternal behavior and stress response in F1 dams were assessed at the first postpartum week. Biochemical analyses and pyrosequencing were used to evaluate the functions of the oxytocin pathway.

The experimental design is shown in Figure 5. Female rats of first-generation (F0) were mated with age-matched male rats (female:male = 2:1) for five days. The vaginal plug was checked every morning, and the day of obtaining a vaginal plug was regarded as gestational day 1. Each male sired 2 litters, which were subjected to either the vehicle or DEHP group. Pregnant rats were housed individually and administered daily with vehicle (*n* = 8) or DEHP (*n* = 8) by oral gavage from gestational days 14 to 21. Second-generation (F1) pups were examined with anogenital distance on postnatal day 1 (PND1) and then were culled to four females and four males in one cage. The F1 female rats were weaned on PND 21 and two female siblings were housed in a cage. The F1 female rats were divided into four groups: two non-exercised groups, including vehicle (C) and DEHP (D), and two exercised groups, including exercised vehicle (Cex) and exercised DEHP (Dex). In the exercised groups, rats were trained to run on a treadmill for 5 weeks. The development of F1 female rats is shown in Supplementary Materials Table S2. The changes in body weight during development showed that there was an effect of DEHP on body weight at 3 weeks of age (F_(1, 44)_ = 5.007, *p* < 0.05, η^2^ = 0.102) and at 4 weeks of age (F_(1, 44)_ = 4.677, *p* < 0.05, η^2^ = 0.096). Additionally, there was an effect of exercise on body weight at 7 weeks of age (F_(1, 44)_ = 6.740, *p* < 0.05, η^2^ = 0.133) and at 8 weeks of age (F_(1, 44)_ = 6.722, *p* < 0.05, η^2^ = 0.133). At 12 weeks of age, F1 female rats were allowed to produce the F2 generation, and the pups were culled to four females and four males in one cage on PND1 as previously mentioned. The F1 female rats that failed to be impregnated during the first mating week were excluded from this study. There was no significant difference in the litter size or sex ratio among groups in F2 offspring, as shown in Supplementary Materials Table S3.

During the first postpartum week, the F1 females were assessed for their maternal behaviors by a pup retrieval test followed by an elevated plus maze to examine their stress responses. The blood and hypothalamus samples from F1 female rats were used for analysis. The levels of oxytocin, BDNF, adrenocorticotropic hormone (ACTH), and glucocorticoid were analyzed by enzyme-linked immunosorbent assay (ELISA). The expression of OTR was analyzed by Western blotting. The levels of hypothalamic *Oxtr* mRNA were analyzed by quantitative real-time polymerase chain reaction (qPCR) and the methylations of the hypothalamic *Oxtr* gene were analyzed by pyrosequencing.

### 4.3. Gestational Administration of DEHP

DEHP (Sigma-Aldrich, St. Louis, MO, USA) was dissolved in corn oil (Sigma-Aldrich, St. Louis, MO, USA) which was prepared fresh every day and treated by oral gavage. The dose of DEHP exposure was 10 mg/kg/day. The control group was supplied with corn oil, and the DEHP group was fed with the same volume of DEHP/corn oil mixture. The estimated DEHP exposure for the adult human population is 1 to 30 µg/kg/day [86]. According to the conversion coefficient, humans are exposed to DEHP doses corresponding to 0.18–2.5 mg/kg/day for exposure in rats [87,88]. The no-observed-adverse-effect level (NOAEL) of DEHP for humans is 48 mg/kg/day, which is converted to an equivalent dose corresponding to 300 mg/kg/day for rats [88]. Therefore, prenatal exposure to DEHP at the dose of 10 mg/kg/day is lower than NOAEL and is considered human-friendly with no known adverse effects.

### 4.4. Treadmill Running

Rats were trained to run on a treadmill at night (19:00–21:00, when the light was off). Initially, rats in the exercised groups were allowed to run on a motor-driven horizontal treadmill (Model Exer 3/6, Columbus Instruments, Columbus, OH, USA), starting at a very low speed and gradually increasing to 8 m/min for 30 min each day for 7 days. Then, the animals were trained for 40 min/day (8 m/min warm-up for 10 min), 7 days/week for 4 weeks. The running speed started at 12 m/min, increased by 3 m/min every week, and reached up to 21 m/min at the end of the training period [43]. The rats were trained on a treadmill without an electric foot shock to reduce stress during treadmill running [89]. In contrast, animals in the non-exercising group were placed on the treadmill without running for 10 min each day for 5 weeks.

### 4.5. Pup Retrieval Test

The pup retrieval test was conducted between 17:00 and 19:00 on postpartum days 3, 5, and 7 according to the reported observational methods [6,90]. Postpartum F1 dams and their pups were brought to the test room in the home cage for 20 min, then the pups were separated from the dam and placed on a heating pad to maintain their body temperature at 37 °C for 30 min. After separation, 8 pups were returned to their home cage in the manner of one male and one female pup placed in each corner. A 30-min video recording began immediately following the return of the pups. During each observation, the following categories of maternal behaviors were recorded by an observer blind to the experimental treatments: the latency to retrieve the first pup (first retrieval latency), the time spent in pup retrieval (retrieval time), the number of pups retrieved (retrieved pups), the time spent on pup licking (licking time), the time spent on arched-back nursing (nursing time), and the time spent in self-grooming of the dam (self-grooming time). The average value from the 3-day observation of the same rat was used for comparison.

### 4.6. Elevated plus Maze

The elevated plus maze was conducted between 17:00 and 19:00 on postpartum day 8. The apparatus consisted of four arms, two opposing open arms (60 cm length × 10 cm width) and two opposite black plastic closed arms (60 cm length × 10 cm width × 40 cm height), joined by a central platform (10 × 10 cm). The four arms were equally illuminated under red light so that the animals did not perceive lighting differences. Each rat was first placed on the central platform facing an open arm, and then its behavior was video recorded for 5 min. All trials were conducted between 17:00 and 19:00, and each rat was tested only once. Ethanol (40% *v*/*v*) was used to clean each arm of the maze between trials to remove odor cues. The number and time of entering into each arm were recorded by an observer blind to the experimental treatments. All four paws inside the arm determined the successful entries. The percentage of open arms entries was calculated by: number of open arms entries (%) = ((number of open arms entries) ÷ (total number of arms entries) × 100%). The following equation calculates the percentage of time spent in open arms: time spent in open arms (%) = ((time spent in open arms) ÷ (exploration time 300 s) × 100%).

### 4.7. Blood and Tissue Sample Collection

After exploration of the elevated plus maze, rats were returned to their home cages and stayed there for 30 min and were sacrificed by 1 min inhalation of CO_2_. The blood samples were collected from the right atria and centrifuged at 1500 rpm for 30 min, then the supernatants were collected and stored at −80 °C. After blood collection, the brain was removed and soaked in ice-cold phosphate-buffered saline (0.05 M Na_2_HPO_4_ and 0.137 M NaCl, pH 7.4) to remove the residual blood, and then the whole hypothalamus was isolated under microscopic observation by dissecting ventral to the thalamus, posterior to the optic chiasma, anterior to the mammillary bodies, and demarcated laterally by the optic tracts. The hypothalamic samples soaked in ice-cold lysis buffer (*n* = 6) were used for Western blot and ELISA. The rest of the hypothalamus was soaked in RNAlater solution (ThermoFisher, Waltham, MA, USA) (*n* = 6) and was used for qPCR and pyrosequencing.

### 4.8. Western Blot

The tissue was homogenized in ice-cold lysis buffer (20 mM Tris, pH 7.5, 150 mM NaCl, 1 mM EDTA, 1 mM EGTA, 1% Triton X-100, 1% deoxycholate, 1 mM sodium fluoride, and 2 mM sodium orthovanadate) and centrifuged at 13,000× *g* for 20 min at 4 °C. Protein in the supernatant was quantified using a BCA Protein Assay kit (ThermoFisher, Waltham, MA, USA) according to the manufacturer’s instructions. Thirty micrograms of protein were mixed with NuPage LDS sample buffer (Invitrogen, Carlsbad, CA, USA) and separated by pre-cast 10% Bis-Tris gel (Invitrogen, Carlsbad, CA, USA) in MOPS running buffer (Invitrogen, Carlsbad, CA, USA) for 50 min at 120 mA and 200 V. Proteins were transferred to a polyvinylidene difluoride membrane (Millipore, Burlington, MA, USA) in NuPage transfer buffer (Invitrogen, Carlsbad, CA, USA) for 60 min at 170 mA and 30 V. After blocking with 5% nonfat milk in TTBS buffer (10 mM Tris, pH 7.5, 150 mM NaCl, and 0.1% Tween 20), the membrane was incubated with primary antibodies specific for each protein for 24 h at 4 °C: rabbit anti-OTR antibody (1:1000, ab217212, Abcam, USA) and mouse anti-actin antibody (1:5000, A2228, Sigma-Aldrich, St. Louis, MO, USA). After washing, the blot was incubated with horseradish peroxidase-conjugated goat secondary antibodies (1:2000, ab97051 and ab97023, Abcam, Eugene, OR, USA) for 60 min at room temperature. The expression of the protein was detected by the enhanced chemiluminescence kit (Invitrogen, Carlsbad, CA, USA) according to the recommended conditions. Digital images of the blots were created by scanning the blots and the optical densities were determined with the Image-Pro Plus software (Media Cybernetics, Rockville, MD, USA). Each protein level was normalized to the control level from the same membrane and presented as the percent of expression (%). Western blot analysis was performed in duplicate, and the average from the same rat was used for comparison.

### 4.9. Enzyme-Linked Immunosorbent Assay

The levels of plasma and hypothalamic oxytocin and BDNF, as well as plasma ACTH and corticosterone, were determined by commercially available assay kits optimized for small volumes, according to the manufacturer’s instructions. The detection limit of each kit for corresponding hormones is 15 pg/mL for oxytocin detection kit (ADI-901-153, Enzo, Farmingdale, NY, USA), 12 pg/mL for BDNF detection kit (ERBDNF, Invitrogen, USA), 6 pg/mL for ACTH detection kit (ab263880, Abcam, USA), and 8.2 pg/mL for corticosterone detection kit (501320, Cayman, USA).

### 4.10. Quantitative Real-Time Polymerase Chain Reaction

RNA was extracted from the hypothalamus using the AllPrep DNA/RNA kit (Qiagen, Valencia, CA, USA) according to the manufacturer’s instructions. One microgram of RNA was processed for cDNA synthesis following the protocol provided in the iScript cDNA Synthesis kit (Bio-Rad, Hercules, CA, USA) and cDNA was then amplified using a 7500 Real-Time PCR System (ThermoFisher, Waltham, MA, USA) and Power SYBR Green Master Mix (ThermoFisher, Waltham, MA, USA). The amplification was performed under the following cycling conditions: (1) 10 min of denaturation at 95 °C; (2) 35 cycles of 15 s at 95 °C and 60 s at 63 °C. All reactions were run in triplicate and their specificity was verified by melting curve analysis. The primer sequences designed for qPCR are shown in the Supplementary Materials Table S4. The comparative Ct measures were used to obtain fold changes (RQ) for the Cex, D, and Dex groups relative to the C group.

### 4.11. Oxtr Methylation Pyrosequencing

Methylation analysis was performed with next generation sequencing (NGS). Genomic DNA was extracted from the hypothalamus by using the AllPrep DNA/RNA kit (Qiagen, Valencia, CA, USA) according to the manufacturer’s instructions. Cell-free DNA (160 ng) was subjected to the bisulfite DNA conversion process using the EZ DNA Methylation-Gold kit (Zymo Research, Freiburg, Germany) according to manufacturer instructions. A region of the *Oxtr* gene corresponding to nucleotide positions 07717398 to 207717832 on chromosome 4 was amplified by PCR using bisulfite-treated DNA as the template [18]. The target *Oxtr* gene was amplified according to the general guidelines of pyrosequencing under the following cycling conditions: (1) denaturation at 95 °C for 10 min; (2) 45 cycles at 95 °C, 54 °C, and 72 °C (each for 30 s); and (3) a final extension cycle at 72 °C for 10 min. The bisulfite specific primer sequences are shown in the Supplementary Materials Table S4. The PCR product with a length of ~400 bp was examined by 2% agarose gel electrophoresis. The NGS library preparation was performed by using Collibri NGS Whole-Genome Library Prep kits (ThermoFisher, Waltham, MA, USA) and VAHTS Multiplex Oligo set 5 Adapters for Illumina (Vazyme, Nanjing, China). The amplicons were purified with VAHTS DNA Clean Magnetic Beads (Vazyme, Nanjing, China) and the size distribution of the amplicons of the adapter-ligated library was checked by the MultiNA MCE-202 with DNA-2500 Kit (Shimadzu, Kyoto, Japan). Paired sequencing was performed by MiSeq sequencer (Illumina, San Diego, CA, USA) with paired 150 bp sequencing reads. The bioinformatics analysis was applied with FASTX-toolkit on Galaxy (www.usegalaxy.org (accessed on 15 October 2018)) and MethTargetedNGS (www.bioconductor.org (accessed 20 October 2018)) version 1.14.0 package with RStudio (Boston, MA, USA).

### 4.12. Statistical Analysis

The statistical analysis was performed with SPSS Statistics (v. 25.0, IBM, Armonk, NY, USA). The two-way ANOVA was used to analyze the data, with DEHP (vehicle and DEHP) and exercise (non-exercise and exercise) as between-subjects factors, and normality was determined using the Shapiro–Wilk normality test. The effect size was shown by partial eta squares (η^2^) as having small (η^2^ = 0.01), medium (η^2^ = 0.06), or large (η^2^ = 0.14) effects. The correlation was used to examine the relationship between Oxtr methylation of each CpG site, *Oxtr* mRNA, and maternal behaviors. Normality was assessed by the Kolmogorov–Smirnov test. Spearman’s rank correlation was used when the data were not normally distributed, whereas Pearson’s correlation was used when the data were normally distributed. All values were expressed as mean ± standard error of the mean (SEM) in the figures. Significance was assumed as *p* < 0.05.

## Figures and Tables

**Table 1:** Effects of prenatal DEHP exposure and childhood exercise on anxiety-like behaviors.

Observed Categories	Groups (Mean ± SEM)				Effect (F Value)	
	C	Cex	D	Dex	DEHP	Exercise
Number of open arms entries (counts)	9.42 ± 1.47	9.92 ± 1.82	8.17 ± 1.81	8.83 ± 1.87	0.444	0.111
Number of close arms entries (counts)	15.33 ± 2.06	15.35 ± 1.91	16.17 ± 2.39	17.25 ± 2.34	0.145	0.002
Number of open arms entries (%)	38.47 ± 3.68	37.83 ± 2.34	31.77 ± 1.88	32.06 ± 2.39	2.336	0.077
Time of open arms entries (s)	67.00 ± 8.03	66.33 ± 9.29	63.25 ± 9.66	64.92 ± 9.22	0.081	0.003
Time of close arms entries (s)	184.50 ± 7.84	190.25 ± 6.38	193.75 ± 8.05	194.50 ± 8.53	0.760	0.176
Time of open arms entries (%)	22.33 ± 2.68	22.11 ± 3.10	21.08 ± 3.22	21.64 ± 3.07	0.081	0.003

C: vehicle control; Cex: exercised vehicle; D: DEHP exposure; Dx: exercised DEHP.

**Table 2:** Effects of prenatal DEHP exposure and childhood exercise on expressions of BDNF and stress-related hormones.

Measured Concentrations	Groups (Mean ± SEM)				Effect (F Value)	
	C	Cex	D	Dex	DEHP	Exercise
Plasma BDNF (pg/mL)	348.72 ± 31.10	375.33 ± 27.92	308.97 ± 24.52	330.01 ± 24.02	2.475	0.777
Hypothalamic BDNF (pg/100 μg protein)	128.26 ± 12.46	131.88 ± 9.27	121.60 ± 9.17	117.63 ± 7.65	1.138	0.000
Plasma ACTH (pg/mL)	115.65 ± 5.11	112.67 ± 4.92	128.96 ± 4.96	121.50 ± 4.79	5.011 *	1.115
Plasma corticosterone (ng/mL)	239.43 ± 22.42	241.72 ± 18.74	303.83 ± 27.73	263.02 ± 28.59	3.009	0.608

C: vehicle control; Cex: exercised vehicle; D: DEHP exposure; Dx: exercised DEHP. *: *p* < 0.05.

## Data Availability

Not applicable.

## Supplementary Materials

The following are available online at https://www.mdpi.com/article/10.3390/ijms22189847/s1.
