# Supplementary material for: Effects of Prenatal Phthalate Exposure and Childhood Exercise on Maternal Behaviors in Female Rats at Postpartum: A Role of Oxtr Methylation in the Hypothalamus
Source: Int J Mol Sci. 2021 Sep 12;22(18):9847. doi: 10.3390/ijms22189847 (PMC8465903; doi:10.3390/ijms22189847)
Supplement: Supplementary file 1 [file ijms-22-09847-s001.zip › ijms-1364405-supplementary.pdf]

Table S1: Correlation between maternal behaviors and *Oxtr* methylation, Table S2: The development of F1 female rats, Table S3: The litter size and sex ratio in F2 offspring, Table S4: Primers sequences.

Supplementary Materials Table S1

Correlation between maternal behaviors and *Oxtr* methylation

		CpG sites											
	Oxtr	**1**	**2**	**3**	**4**	**5**	**6**	**7**	**8**	**9**	**10**	**11**	**12**
***Oxtr* expression**	1.00	−0.31	−0.30	−0.29	−0.28	−0.22	−0.16	0.10	−0.10	−0.43*	−0.27	−0.16	−0.35
**First retrieval latency**	-0.63***	0.38	0.28	0.40	0.45*	0.05	0.03	−0.25	0.24	0.32	0.35	0.19	0.35
**Retrieval time**	0.61***	−0.24	−0.10	−0.26	−0.31	−0.14	−0.20	0.14	−0.06	−0.30	−0.25	−0.11	−0.25
**Retrieved pups**	0.37	0.04	0.13	0.06	−0.20	−0.09	−0.16	0.00	−0.11	−0.21	0.08	−0.07	−0.12
**Licking time**	0.62***	−0.38	−0.39	−0.25	−0.39	−0.27	−0.21	−0.05	−0.29	−0.43*	−0.26	−0.31	−0.38
**Nursing time**	−0.31	−0.15	−0.09	0.02	−0.26	−0.14	-0.07	−0.21	−0.30	−0.08	−0.02	−0.11	−0.15
**Self-grooming time**	0.08	0.08	0.11	0.20	−0.07	0.03	0.12	0.03	0.03	0.07	0.13	0.19	0.08

		CpG sites											
	**13**	**14**	**15**	**16**	**17**	**18**	**19**	**20**	**21**	**22**	**23**	**24**	**25**
***Oxtr* expression**	−0.35	−0.36	−0.33	−0.41*	−0.45*	−0.34	−0.32	0.10	0.05	−0.19	−0.04	0.15	−0.01
**First retrieval latency**	0.23	0.39	0.31	0.52*	0.46*	0.34	0.20	−0.05	0.07	0.21	0.12	−0.23	−0.11
**Retrieval time**	−0.25	−0.28	−0.28	−0.34	−0.27	−0.30	-0.41*	0.08	−0.15	−0.13	−0.02	0.17	0.03
**Retrieved pups**	−0.17	−0.05	−0.23	−0.21	−0.17	−0.22	−0.13	−0.06	−0.04	−0.18	−0.21	0.09	0.08
**Licking time**	−0.34	−0.29	-0.47*	-0.57***	-0.61***	-0.46*	−0.16	−0.04	0.00	−0.37	−0.14	0.31	0.17
**Nursing time**	0.01	0.11	−0.17	−0.13	−0.07	−0.19	−0.03	0.13	−0.10	−0.23	−0.26	0.03	0.17
**Self-grooming time**	−0.19	0.21	0.00	−0.02	−0.17	−0.25	-0.08	−0.10	0.07	−0.15	0.04	0.34	0.29

-1.0 0 1.0
Correlation coefficient

Supplementary Materials Table S2

The development of F1 female rats

**Table S2: Effects of prenatal DEHP exposure and childhood exercise on body weight of F1 females**						
Observed categories	**Groups (Mean ± SEM)**				**Effect (F value)**	
	**C**	**Cex**	**D**	**Dex**	**DEHP**	**Exercise**
Postnatal 3 weeks	40.26±0.96	40.35±1.03	38.69±0.34	38.57±0.36	5.007*	0.000
Postnatal 4 weeks	60.16±0.96	61.43±0.84	59.53±0.85	58.38±0.75	4.677*	0.006
Postnatal 5 weeks	95.66±2.34	93.71±1.95	92.53±1.45	91.79±1.20	1.978	0.564
Postnatal 6 weeks	140.82±3.02	135.37±2.84	136.73±3.07	132.13±3.57	1.366	2.566
Postnatal 7 weeks	180.70±3.11	172.40±3.21	178.04±2.70	171.07±2.70	0.460	6.740*
Postnatal 8 weeks	235.66±2.92	224.38±4.08	231.33±4.02	222.66±4.23	0.616	6.722*

C: vehicle control; Cex: exercised vehicle; D: DEHP exposure; Dx: exercised DEHP
*: $p < 0.05$

Supplementary Materials Table S3

The litter size and sex ratio in F2 offspring

Table S3: Effects of prenatal DEHP exposure and childhood exercise on sex ratio of F2 offspring						
Observed categories	Groups (Mean ± SEM)				Effect (F value)	
	C	Cex	D	Dex	DEHP	Exercise
Number of male offspring	6.08±0.36	5.75±0.35	5.83±0.32	5.50±0.38	0.501	0.891
Number of female offspring	6.50±0.23	5.75±0.39	6.58±0.29	6.17±0.32	0.637	3.466
Male-to-female ratio	0.93±0.04	1.07±0.11	0.91±0.07	0.91±0.07	1.385	0.825
C: vehicle control; Cex: exercised vehicle; D: DEHP exposure; Dx: exercised DEHP						

Supplementary Materials Table S4

Primers sequences

Primer Name	Primer Sequence
**Primers for RT-PCR**	
Forward	5’-CTGGATATGCGCAAGTGTCTTC-3’
Reverse	5’-GAAGGAAGCGCCCTAAAGGTAT-3’
**Bisulfite specific primers**	
Oxtr_rn_CpG1-17_BSP-255bp-F	5’-GGAAGTTGTATTGTAGTGGATGGTTTT-3’
Oxtr_rn_CpG1-17_BSP-255bp-R	5’-AATACTACAACCTCCRACTCCAACTATAA-3’
Oxtr_rn_CpG10-25_BSP-291bp-F	5’-GGATGTYGTAGGAAGATAAGAGAGAGTT-3’
Oxtr_rn_CpG10-25_BSP-291bp-R	5’-CACTTTCCAAAATTCCAATATCTCTAAA-3’
Illumina primer overhang universal adapter	5’-AATGATACGGCGACCACCGAGATCTACACTCTTTCC CTACACGACGCTCTTCCGATCT-3’
Illumina primer overhang index adapter	5’-GATCGGAAGAGCACACGTCTGAACTCCAGTCAC-XX XXXXXX-ATCTCGTATGCCGTCTTCTGCTTG-3’
